# Roles of Putative Type II Secretion and Type IV Pilus Systems in the Virulence of Uropathogenic *Escherichia coli*

**DOI:** 10.1371/journal.pone.0004752

**Published:** 2009-03-09

**Authors:** Ritwij Kulkarni, Bijaya K. Dhakal, E. Susan Slechta, Zachary Kurtz, Matthew A. Mulvey, David G. Thanassi

**Affiliations:** 1 Center for Infectious Diseases, Department of Molecular Genetics and Microbiology, Stony Brook University, Stony Brook, New York, United States of America; 2 Department of Pathology, Division of Cell Biology and Immunology, University of Utah, Salt Lake City, Utah, United States of America; Theodor-Boveri-Institut fur Biowissenschaften, Wurzburg, Germany

## Abstract

**Background:**

Type II secretion systems (T2SS) and the evolutionarily related type IV pili (T4P) are important virulence determinants in many Gram-negative bacterial pathogens. However, the roles of T2SS and T4P in the virulence of extraintestinal pathogenic *Escherichia coli* have not been determined.

**Methodology/Principal Findings:**

To investigate the functions of putative T2SS and T4P gene clusters present in the model uropathogenic *E. coli* (UPEC) strains UTI89 and CFT073, we deleted the secretin gene present in each cluster. The secretin forms a channel in the outer membrane that is essential for the function of T2S and T4P systems. We compared the secretin deletion mutants with their wild type counterparts using tissue culture assays and the CBA/J mouse model of ascending urinary tract infection. No deficiencies were observed with any of the mutants in adherence, invasion or replication in human bladder or kidney cell lines, but UTI89 Δ*hofQ* and UTI89 Δ*gspD* exhibited approximately 2-fold defects in fluxing out of bladder epithelial cells. In the mouse infection model, each of the knockout mutants was able to establish successful infections in the bladder and kidneys by day one post-infection. However, UTI89 Δ*hofQ* and a CFT073 Δ*hofQ *Δ*yheF* double mutant both exhibited defects in colonizing the kidneys by day seven post-infection.

**Conclusions/Significance:**

Based on our results, we propose that the putative T4P and T2S systems are virulence determinants of UPEC important for persistence in the urinary tract, particularly in renal tissues.

## Introduction

*Escherichia coli* are a diverse group of Gram-negative bacteria that can be divided into three major subgroups: commensals in the intestinal tract of warm-blooded mammals, agents of intestinal infections such as hemmorhagic colitis and hemolytic-uremic syndrome, and extra-intestinal pathogenic *E. coli* (ExPEC) [1]. ExPEC are causative agents of a range of diseases, including urinary tract infections (UTIs), nosocomial pneumonia, neonatal meningitis, and neonatal sepsis. UTIs comprise a range of disorders, including cystitis (infection of the bladder) and pyelonephritis (infection of the kidney), which are defined by the presence of microorganisms in the otherwise sterile urinary tract. UTIs are one of the most common bacterial infections, with an estimated 33% of women in the United States suffering from a UTI requiring antimicrobial therapy by the age of 24 and 40–50% of women experiencing a UTI at least once during their lifetime [2], [3]. Even though UTIs are associated with minimal morbidity, the annual financial costs associated with their incidence are estimated at a staggering 2 billion dollars for the US alone [2], [3].

There are many known microbial causative agents of UTIs, including *Candida albicans*, *Pseudomonas aeruginosa*, *Staphylococcus saprophyticus*, and *E. coli*. Of these, uropathogenic *E. coli* (UPEC) are of special interest because they cause approximately 40% of hospital and 80% of community acquired UTIs [4]. The success of UPEC as pathogens can be attributed to their panoply of virulence factors. These include a variety of toxins, such as cytotoxic and necrotizing factor 1 (CNF1), hemolysin (*hlyA*), and secreted autotransporter toxin (Sat), and a diverse range of adhesins, including type 1 and P pili. CNF1 is a dermonecrotic toxin that increases the invasiveness of UPEC by activating Rho GTPases [5] and also modulates the function(s) of polymorpho-nuclear leukocytes (PMNs) [6]. HlyA targets erythrocytes and leukocytes to bring about cell lysis [1], but exactly how it aids UPEC virulence is unknown [6]. Sat is a cytolytic vacuolating toxin that has been shown to damage the glomerular membrane and proximal tubule cells in the kidney, and to create lesions in intestinal and renal epithelial cell tight junctions [7], [8]. The adhesins present at the tips of type 1 and P pili, FimH and PapG, respectively, aid UPEC in adhering to the bladder and kidney epithelial cells of the host [9], [10]. Moreover, FimH behaves as an invasin, causing reorganization of the actin cytoskeleton and localized alterations in the host cell membrane [11]. Thus, a variety of structurally and functionally different virulence factors work to aid the pathogenesis of UPEC in the host urinary tract.

Until recently, UPEC were considered to be predominantly extracellular pathogens of the urinary tract. Even though there were reports showing UPEC internalization by mouse bladder superficial epithelial cells, this was considered a result of phagocytosis by the bladder epithelium [12]. In 1998, it was shown that UPEC adhere to and invade cells of the bladder epithelium in a FimH-dependent manner [9]. Once internalized, the bacteria escape the endosomes by an unknown mechanism and start replicating in the host cytoplasm to form biofilm-like structures termed intracellular bacterial communities (IBCs). The growing IBCs push against the epithelial cell membrane giving it a pod-like appearance [13]. A recent study found that by 1 h post infection with 10^7^ colony forming units (CFU) of the UPEC strain UTI89, approximately 50% of the bacteria in the murine bladder were intracellular, and by 6 h post infection 20 to 210 IBCs per bladder were detected [14]. In response to replicating bacterial loads, epithelial cells exfoliate and are purged by urine flow as part of an innate immune response. PMNs are also recruited to sites of UPEC infection. Intracellular niches are considered to provide the UPEC a safe haven away from the host's immune defenses. UPEC escape the exfoliating cells by fluxing out and invading the underlying layers of cells [9], [15].

Type II secretion systems (T2SS) are involved in the secretion of many important effectors in diverse Gram-negative pathogenic bacteria [16], [17]. For example, the secretion of cholera toxin is mediated by the Eps T2SS in *Vibrio cholerae* [18] and enterotoxigenic *E. coli* (ETEC) employ a T2SS to secrete heat labile enterotoxin (LT) [19]. The entire T2SS apparatus, termed the secreton [20], spans the bacterial envelope. Structurally as well functionally, the components of the secreton form three subassemblies: an outer membrane (OM) channel formed by the secretin [21], a platform of proteins associated with the cytoplasmic membrane [22], and a set of proteins that are proposed to assemble into a pilus-like structure (the pseudopilus) in the periplasm [23]. The secretin is a member of a family of gated pores that also includes proteins involved in type IV pilus (T4P) biogenesis, filamentous phage extrusion, and the type III secretion system [24]. The pseudopilus is proposed to propel effector molecules across the OM through the secretin by undergoing cycles of extension and retraction within the periplasm [23]. The cytoplasmic membrane platform of proteins is thought to act as an assembly point for the pseudopilus and to provide energy to drive the secretion process [22].

The components of the T2SS are similar to the evolutionarily related T4P system. T4P are adhesive appendages on the bacterial surface that perform a variety of functions, including twitching motility, adhesion, biofilm formation, and horizontal genetic transfer [25], [26]. *E. coli* K12, the common laboratory strain, possesses gene clusters coding for a T2SS as well as for T4P biosynthesis. The K12 T2SS and T4P appear to be non-functional under standard culture conditions, although the T2SS was shown to mediate the secretion of chitinase in the absence of the nucleoid structuring protein H-NS [27] and the T2SS secretin gene was found to be required for surface expression of the DraD invasin [28]. The putative major pilin of the K12 T4P, PpdD (prepilin peptidase dependent protein D), could be assembled into pili when ectopically expressed in *Pseudomonas aeruginosa* [29] and *Klebsiella oxytoca* [30]. In addition, recent studies demonstrated that enterohemorrhagic *E. coli* (EHEC) O157∶H7 expresses functional T4P composed of PpdD when grown on minimal casein medium [31], [32]. Both T2SS and T4P have well-studied roles in the virulence of intestinal pathogenic *E. coli*. ETEC secretes heat labile enterotoxin via a T2SS [19] and employs T4P (longus pili) as a colonization factor [33]. Enteropathogenic *E. coli* (EPEC) use T4P (bundle-forming pili) for localized adherence in the intestinal tract and autoaggregation [34]. In contrast to these established functions in intestinal pathogenic *E. coli*, roles for T2SS and T4P in the virulence of ExPEC have not been established.

In this study, we investigated the roles of the putative T2S and T4P systems in the pathogenesis of the model UPEC strains CFT073 and UTI89, isolated from patients suffering from pyelonephritis and cystitis, respectively [35], [36]. We constructed knockout mutations in OM secretin genes to disable the T2SS and T4P clusters present in CFT073 and UTI89, and compared the mutant and wild-type (WT) strains under *in vitro* and *in vivo* conditions. Our findings identify the *hofQ* secretin as a virulence factor of UTI89, and *hofQ* and/or the *yheF* secretin as virulence determinants of CFT073. In particular, our results show that these systems are important for persistent infection of the upper urinary tract.

## Results

### T2SS and T4P gene clusters in CFT073 and UTI89

**Figure 1:**
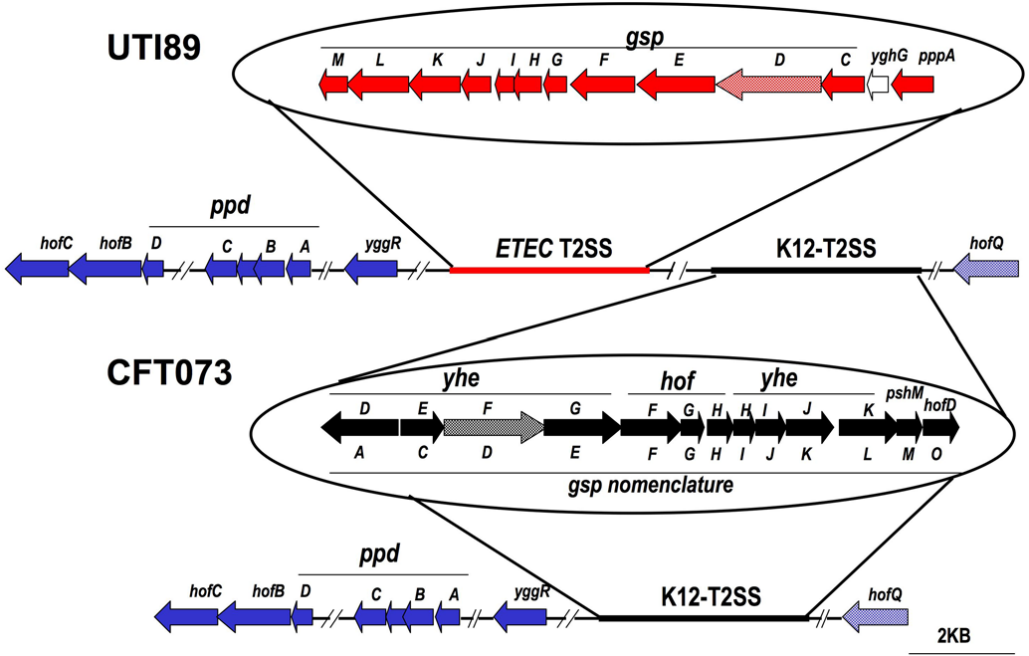
Schematic Representation of the T2SS and T4P gene clusters in UTI89 and CFT073. The *E. coli* K12 and ETEC homologous T2SS gene clusters are shown in black and red, respectively. T4P genes are shown in blue. For the K12-T2SS, the *E. coli* K12 gene names are listed above the arrows and the generic *gsp* nomenclature is given below. The OM secretin genes are indicated by the hatched arrows. The open arrows are genes that do not show homology to any of the known T2SS or T4P genes.

UPEC strain CFT073 is a well characterized strain isolated from the blood of a woman suffering from pyelonephritis [35], whereas UTI89 is a UPEC strain isolated from a cystitis patient [36]. Annotated complete genome sequences for both these model clinical isolates have been published (CFT073, accession NC_00431; UTI89, accession NC_007946) [37], [38]. The T2SS and T4P genes present in the UTI89 and CFT073 genomes are shown schematically in Figure 1. Both UPEC clinical isolates possess gene clusters homologous to the putative T2SS gene cluster present in *E. coli* K12 (K12-T2SS). Both UPEC strains also possess orthologs of the putative T4P genes present in *E. coli* K12. The T2SS and T4P genes common to both UTI89 and CFT073 have very high amino acid homology to the corresponding *E. coli* K12 systems (91–99% identity, except for the *gspI* minor prepilin, which has 84% identity). In addition to these K12-homologous genes, UTI89 possesses a second putative T2SS gene cluster that has 84–97% amino acid homology to a cluster present in ETEC strain H10407 (ETEC-T2SS). The ETEC-T2SS has relatively low homology to the K12-T2SS (16–60% amino acid identity). The ETEC-T2SS cluster is not present in strain CFT073. Nonsense mutations, insertions, or other disruptions are not present in any of the T2SS and T4P gene sequences in UTI89 and CFT073, indicating that these systems are intact and likely to be functional.

To analyze the importance of the putative T2SS and T4P gene clusters in the pathogenesis of UPEC, we targeted the OM secretin of each system for deletion mutagenesis using the bacteriophage λ Red recombinase system [39]. The secretin component forms a gated channel required for export of T2SS effectors or T4P across the OM. We made single knockout mutations of each of the three secretins present in UTI89, namely *yheF* for the K12-T2SS cluster, *gspD* for the ETEC-T2SS cluster, and *hofQ* for the T4P cluster (Fig. 1). For CFT073, we constructed single knockout mutations of the *yheF* and *hofQ* secretins, as well as a double deletion strain lacking both secretins (Fig. 1). Proper construction of the knockout mutations was verified by PCR (data not shown) using primers specific for the flanking regions of the targeted genes (Table 1). We also checked each of the knockout mutants for growth in LB, M63 minimal medium, and in RPMI supplemented with 10% FBS; growth of each mutant was similar to the parental WT strain (data not shown).

**Table 1:** Primers used in this study.

Primer	Sequence	Size of PCR Product (bp)	
		WT	Knockout
T4P Secretin (*hofQ*)			
Knockout forward	TTACTCACTGGAAACCAGTCGTGGCGTGATAAACACCACTAACTCGCGTCGTGTAGGCTGGAGCTGCTTC		
Knockout reverse	ATGAAGCAATGGATAGCCGCACTACTGTTGATGCTGATACCCGGCGTACACATATGAATATCCTCCTTAGT		
Flank forward	CATATTGAGTTGTTGAGCTAACTGG	1724	726
Flank reverse	GACAATTTTACAGCTGACGCC		
RT-PCR forward	GATGGTGGATGACGTTCCG	650	
RT-PCR reverse	AACGTCCGTTGATGCGTCC		
Clone forward	CAACAAGCTTGTGAGCCGTTTTTCGTTAAGCGTGG (HindIII)		
Clone reverse	GATAAAGCTTCATCATGGAACGCAGGCAGC (HindIII)		
ETEC-T2SS secretin (*gspD*)			
Knockout forward	TTAACGCGTTCTCCCGGCATTGAGGAACGCGCGTACTTCCGGCGGTAAGGTGTGTAGGCTGGAGCTGCTT		
Knockout reverse	GTGTTTTGGCGTGATATGACGTTGTCTATCTGGCGTAAGAAGACAACTGGCATATGAATATCCTCCTTA		
Flank forward	AGAGAGCGACAACGGATGAACATGG	2343	323
Flank reverse	CGCCATTGCGCTAAATCAGC		
RT-PCR forward	CATACCGTCACGCAAAATGGTC	899	
RT-PCR reverse	CCAGCAAACACAGTAATGCCCTG		
K12-T2SS secretin (*yheF*)			
Knockout forward	GGACTCAATAAAATCACCTGCTGCTTGCTGGCAGCACTACTCATGCCTTGTGTGTAGGCTGGAGCTGCTT		
Knockout reverse	TCACCGTGACGATGGCGCAGGAGCGTGACTGTTGAACGTGTTCTCATCCACATATGAATATCCTCCTTA		
Flank forward	GAACTTCAGGAATGAATGG	2018	194
Flank reverse	TGAATTCTCATAAGAATGCC		
RT-PCR forward	TGCAGGACACGCTGAGAACG	350	
RT-PCR reverse	GAACGTTCTCAAGCGGTACG		
Clone forward	TGCTAAGCTTACAATTCGTCGCAACGGC (HindIII)		
Clone reverse	AGCGGGATCCTGGCGGGGTACGGTGAGTG (BamHI)		
*16S rRNA*			
Forward	TGACGGGGGCCCGCACAAGC	222	
Reverse	CGGCCGGACCGCTGGCAACA		

The restriction sites introduced in the cloning primers are indicated in parentheses and their location within the primer sequence is underlined.

### Roles of the UPEC secretin genes in interactions with bladder and kidney epithelial cells

The functions of the secretins belonging to the putative T2SS and T4P gene clusters in UTI89 and CFT073 were first assessed using a modified gentamicin protection assay [11] (see Methods). This assay tests the ability of UPEC to adhere to and invade human urothelial cells, form intracellular replicating foci, and efflux out of the infected cells. The assay was carried out using the 5637 human bladder and A498 human kidney cell lines. We observed no defects for the CFT073 or UTI89 secretin knockout mutants in adhering to or invading the human bladder or kidney cells compared to the parental WT strains (data not shown). In addition, no differences were detected in the ability of the knockout mutants to grow intracellularly in the bladder or kidney cells, over a period of 48 hours.

**Figure 2:**
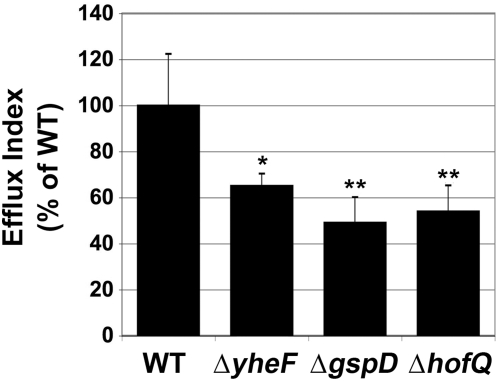
Efflux of UTI89 WT and secretin mutant strains from 5637 human bladder epithelial cells. A modified gentamicin protection assay (see Materials and Methods) was used to calculate efflux of WT UTI89 or the indicated deletion mutant from monolayers of 5637 human bladder epithelial cells. Cell lines were infected with bacteria the previous day and incubated overnight in the presence of gentamicin to kill extracellular bacteria. Cells were then incubated for 6 h in the absence of gentamicin. The efflux index was calculated by dividing the number of bacteria released into the medium after the 6 h incubation without gentamicin by the intracellular bacteria present after the overnight incubation. The efflux index for each strain was normalized to the WT strain. The UTI89 Δ*gspD* and Δ*hofQ* mutants consistently exhibited significantly reduced efflux index values compared to bladder cells infected with WT UTI89. Efflux index values for the UTI89 Δ*yheF* mutant were not always reduced compared to WT. Each deletion mutant was compared with the WT strain at least 3 times. Values are shown as a percentage of WT; error bars indicate SD; *, p<0.05; **, p<0.01.

Bladder epithelial cells are exfoliated and washed off by urine as an innate immune response to infection by UPEC [15]. To escape the exfoliating cells, UPEC efflux out of the infected cells and spread to other parts of the urinary tract. Interestingly, the UTI89 Δ*hofQ* and Δ*gspD* strains consistently exhibited approximately 2-fold reductions (p<0.01) in their ability to efflux out of human bladder cells as compared to the WT UTI89 strain (Fig. 2). The UTI89 Δ*yheF* mutant also exhibited reduced efflux in some experiments (Fig. 2), but in experimental replicates, this mutant was not consistently defective compared to the WT strain. The efflux defect was specific to the UTI89 cystitis isolate, as none of the CFT073 secretin knockout mutants exhibited defects in fluxing out of the bladder cells (data not shown). Furthermore, the efflux defect occurred only in bladder cells, as each of the UTI89 and CFT073 secretin knockout mutants fluxed out of kidney cells similar to the parental WT strains (data not shown). Thus, both the putative T2S and T4P systems appear to assist the escape of the UTI89 cystitis isolate, but not the CFT073 pyelonephritis isolate, from bladder epithelial cells. Attempts to rescue the efflux defects of the UTI89 mutants by expressing the deleted secretin in trans from expression plasmids were not successful, and thus we cannot rule out that the observed defects were caused by secondary effects of the deletion mutations. However, the complemented strains exhibited even greater efflux defects compared to the uncomplemented mutants (data not shown), suggesting that our inability to complement the deletions may have been due to constitutive expression of the secretins from an artificial promoter.

UPEC suppresses the induction of IL-6, a pro-inflammatory and immunomodulatory cytokine, produced by the urothelium in response to bacterial LPS and IL-1 [40]. One hypothesis explaining the mechanism behind the cytokine-suppression is that UPEC secretes effectors that downregulate host cell NF-κB signaling to repress transcription of pro-inflammatory and apoptotic genes [40]. To analyze whether the putative T2SS and/or T4P gene clusters are involved in the secretion of this unknown effector, we compared the different UTI89 and CFT073 secretin knockout mutants with their parental WT strains for ability to suppress IL-6 production in response to LPS by the 5637 human bladder cell line. However, the knockout mutants were able to suppress IL-6 formation just as well as the WT strains (data not shown). We also tested whether the UPEC secretins might be involved in the secretion of a toxic effector molecule. The UTI89 and CFT073 knockout mutants were compared with their parental WT strains for differences in cytotoxicity toward the 5637 human bladder cell line. None of the mutants exhibited decreased cytotoxicity, as measured by LDH release during a 3-hour infection assay (data not shown). In addition, no differences were detected between WT UTI89 and the secretin knockout mutants in the amount of LDH released during the efflux assay carried out as described in Materials and Methods. This indicates that a defect in secretion of a cytotoxic effector molecule is not responsible for the decreased efflux of the UTI89 Δ*hofQ* and Δ*gspD* strains from bladder epithelial cells.

### Roles of the UPEC secretin genes in pathogenesis

**Figure 3:**
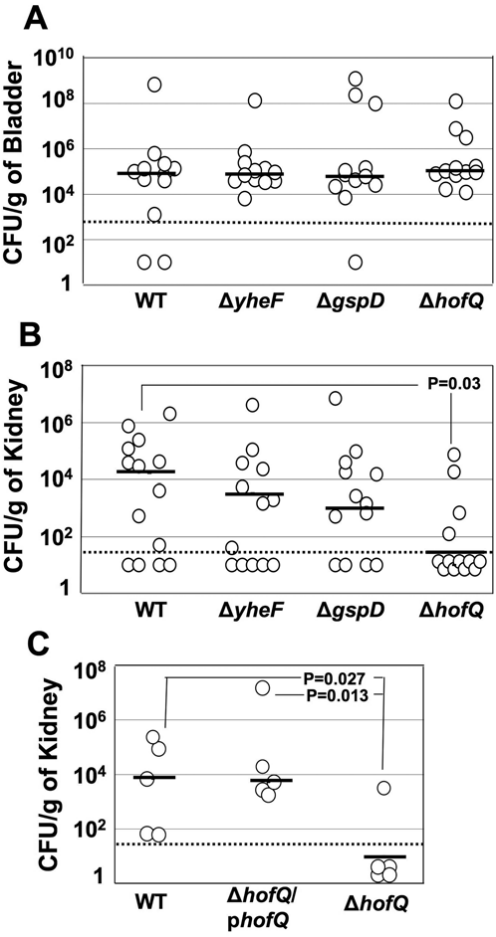
Mouse infection experiments with UTI89. Eight week-old, female CBA/J mice were infected by trans-urethral catheterization with the indicated WT or secretin knockout strains of UTI89. Bladders and kidneys were harvested on day 7 post-infection, weighed, homogenized and CFU/g of organ weight determined. The UTI89 Δ*hofQ* mutant was consistently defective in colonizing kidneys (p = 0.03) (B), whereas no differences were observed in CFU recovered from the bladder (A). The kidney colonization defect of UTI89 Δ*hofQ* (p = 0.027) was rescued by reintroduction of *hofQ* in trans (p = 0.013) (C). The bars indicate median CFU values. The limit of detection is indicated by the dotted line. In panels (A) and (B), the data from two independent experiments were combined together. Panel (C) shows data from one experiment. P values were calculated by Mann-Whitney test for non-parametric data with one-tailed P value.

**Figure 4:**
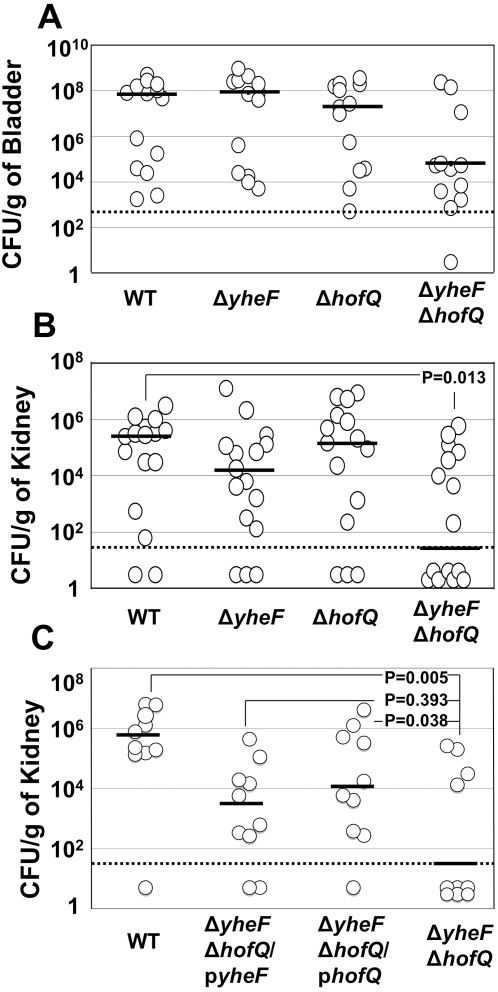
Mouse infection experiments with CFT073. Eight week-old, female CBA/J mice were infected by trans-urethral catheterization with the indicated WT or secretin knockout strains of CFT073. Bladders and kidneys were harvested on day 7 post-infection, weighed, homogenized and CFU/g of organ weight determined. The CFT073 Δ*yheF* Δ*hofQ* double mutant was significantly defective in colonizing the kidneys (p = 0.013) (B), while bladder CFU (A) were lowered as compared to WT, but this difference was not significant. The kidney colonization defect of the CFT073 Δ*yheF* Δ*hofQ* double mutant (p = 0.005) was restored to WT-like levels by plasmid-borne *hofQ* (p = 0.038) (C). Addition of *yheF* in trans also increased colonization levels, but this was not statistically significant (p = 0.393) (C). The bars indicate median CFU values. The limit of detection is indicated by the dotted line. In panels (A) and (B), the data from two independent experiments were combined together. Panel (C) shows data from one experiment. P values were calculated by Mann-Whitney test for non-parametric data with one-tailed P value.

To determine the roles of the putative T2SS and T4P gene clusters in the ability of UPEC to successfully infect the urinary tract, we tested the UTI89 and CFT073 secretin knockout mutants using the CBA/J mouse model of ascending UTI [41]. For UTI89, we inoculated 10^7^ CFU of the WT strain or one of the secretin mutants into the bladder by trans-urethral catheterization. At days 1 and 7 post-infection, animals were sacrificed and CFU/g of bladder or kidney were determined. All knockout mutants colonized the bladder and kidneys at levels similar to WT UTI89 on day 1 post-infection (data not shown). All knockout mutants also colonized the bladder similar to the WT strain on day 7 post-infection (Fig. 3A). In contrast, the UTI89 Δ*hofQ* mutant was significantly defective in colonizing the kidneys on day 7 post-infection (p = 0.03, Fig. 3B). This defect was specific to the Δ*hofQ* mutant, as the Δ*yheF* and Δ*gspD* mutants exhibited no significant defects in kidney colonization (Fig. 4B). Complementation of the UTI89 Δ*hofQ* mutant with plasmid p*hofQ* (expressing WT *hofQ*) restored colonization of renal tissue in the CBA/J mouse infection model to levels similar to the WT strain (Fig 3C). These results define a specific function for the *hofQ* secretin, which belongs to the putative T4P system, in the ability of UTI89 to colonize the kidney.

We next performed similar experiments with the CFT073 secretin mutants. 10^7^ CFU of WT CFT073, the Δ*yheF* or Δ*hofQ* single secretin mutant, or the double secretin mutant (Δ*yheF* Δ*hofQ*) were inoculated into the bladder of CBA/J mice by trans-urethral catheterization. As found for UTI89, none of the CFT073 mutants showed defects in colonizing the bladder or kidneys compared to WT CFT073 on day 1 post-infection (data not shown). The single secretin knockout mutants of CFT073 also showed no significant defects in colonizing the urinary tract on day 7 post-infection (Fig. 4A and B). However, bacterial titers recovered from kidneys on day 7 post-infection of mice infected with the CFT073 Δ*yheF* Δ*hofQ* double mutant were significantly lower (p = 0.013) compared to WT CFT073 (Fig. 4B). In addition, we noted consistently reduced, though not statistically significant, median bacterial titers recovered from the bladders of mice infected with the double knockout strain (Fig. 4A). To confirm the specificity of the kidney phenotype for the CFT073 Δ*yheF* Δ*hofQ* double mutant, we complemented this strain with either *yheF* or *hofQ* in trans, using plasmids p*yheF* or p*hofQ*, respectively. Complementation with p*hofQ* significantly restored kidney colonization (Fig. 4C). Complementation with p*yheF* also increased bacterial colonization in the kidney, but this did not reach statistical significance (Fig. 4C). These results demonstrate a specific role for *hofQ*, belonging to the putative T4P system, and likely also *yheF*, belonging to the putative K12-T2SS in the ability of CFT073 to persist within the urinary tract, particularly in the kidneys.

## Discussion

Many Gram-negative bacterial pathogens use the T2SS and the evolutionarily related T4P system to secrete and/or assemble virulence factors. Although functions for these secretion pathways have been established for intestinal pathogenic *E. coli*, their roles in the virulence of ExPEC have not been investigated. In this report, we begin to address this question by constructing deletion mutations to disable putative T2SS and T4P gene clusters present in the genomes of the model UPEC strains UTI89 and CFT073. We compared these mutants to their WT counterparts using both in vitro and in vivo assays. Our results show that putative T4P and/or T2S systems are important to the pathogenesis of UPEC in the urinary tract, particularly in the ability to successfully colonize and persist within the kidneys.

Both the cystitis isolate UTI89 and the pyelonephritis isolate CFT073 possess putative T2SS and T4P gene clusters sharing very close homology (generally 91–99% amino acid identity) with genes present in *E. coli* K12. In K12 strains, the T2SS does not appear to be expressed under standard growth conditions, but this system was shown to be de-repressed in mutants lacking the nucleotide structuring protein H-NS and to function in the secretion of chitinase [27]. Similarly, the *E. coli* K12 T4P genes are transcribed at low levels and T4P are not detected under standard laboratory conditions [29]. Indeed, we were unable to detect expression of T4P by the UPEC strains grown under a variety of conditions using whole-bacteria, negative-stain electron microscopy (R.K. and D.G.T., unpublished data). We note that a recent study showed that the major pilin subunit PpdD (HcpA) was assembled into T4P in EHEC when the bacteria were cultured on minimal casein medium [31]. Furthermore, expression of the *ppdDhofBC* (*hcpABC)* gene cluster from EHEC in an *E. coli* K12 strain resulted in surface expression of the PpdD pilin, suggesting that the T4P assembly machinery in K12 strains is functional [32]. We were also unable to detect differences in proteins secreted into culture supernatant fractions between the secretin knockout mutants and the parental UPEC strains (R.K. and D.G.T., unpublished data), and thus could not confirm functionality of the T2SS gene clusters. However, the *yheF* (*gspD*) secretin was recently found to be required for surface expression of the DraD invasin in both uropathogenic and laboratory *E. coli* strains [28], suggesting that the T2SS gene cluster is functional. Finally, the virulence defects we observed for the UTI89 Δ*hofQ* and CFT073 Δ*yheF* Δ*hofQ* mutants support the idea that the K12-homologous T2SS and T4P gene clusters are functional and expressed under conditions encountered within the host.

In addition to the K12-homologous systems, the UTI89 cystitis isolate contains a second putative T2SS cluster homologous to genes present in ETEC. In ETEC this T2SS enables the secretion of heat labile enterotoxin, which is critical for pathogenesis in the intestinal tract [19]. This raises the possibility that the ETEC-T2SS might allow secretion of a toxin or other virulence factor by UTI89. Furthermore, since the ETEC-T2SS is absent from the genome of the CFT073 pyelonephritis isolate, the ETEC-T2SS might be specifically associated with the ability of UTI89 to cause cystitis. To check for a correlation between the cystitic nature of a UPEC strain and the presence of the ETEC-T2SS, we analyzed a panel of pathogenic and non-pathogenic *E. coli* isolates by PCR using ETEC-T2SS specific primer pairs. Results from this analysis indicated that the ETEC-T2SS gene cluster is widespread among *E. coli* clinical isolates, without any apparent correlation with the origin of the isolate (R.K. and D.G.T., unpublished data). Examination of the available genome sequences of *E. coli* strains by BLAST analysis also showed the ETEC-T2SS to be prevalent. Thus, CFT073 appears to be an outlier among *E. coli* strains in lacking the ETEC-T2SS.

In the CBA/J mouse model of ascending UTI, we found that the Δ*hofQ* mutant of UTI89 was consistently defective in colonization of kidneys on day 7 post-infection as compared to WT UTI89. Complementation of UTI89 Δ*hofQ* with plasmid-borne *hofQ* restored kidney colonization, confirming that the defect of the knockout mutant was specifically due to loss of the *hofQ* secretin. Similar in vivo defects were not observed for the UTI89 Δ*yheF* or Δ*gspD* mutants. If *hofQ* is indeed involved the expression of T4P, these observations reveal a specific role for T4P in the ability of UTI89 to persistently colonize the kidney. In the pyelonephritogenic isolate CFT073, mutants containing single deletions of Δ*hofQ* or Δ*yheF* were able to infect and colonize bladder and kidney tissues similar to the WT strain. However, a CFT073 double knockout mutant lacking both *yheF* and *hofQ* showed significantly reduced colonization of the kidney on day 7 post-infection. The CFT073 double mutant also exhibited reduced bacterial titers in the bladder on day 7 post-infection, although this reduction was not statistically significant. These results suggest that both the putative T2S and T4P systems contribute to the ability of CFT073 to persistently colonize the urinary tract. The two secretins, *yheF* and *hofQ*, from CFT073 may perform overlapping or redundant functions, explaining why a defect was only detected in the double knockout strain. Complementation of the CFT073 double mutant with *hofQ* in trans significantly restored kidney colonization, but the increased colonization upon expression of *yheF* in trans did not reach statistical significance. This could indicate a more important role for *hofQ* in colonization of the kidney, in agreement with the UTI89 results. Alternatively, lack of full complementation by *yheF* could be attributed to expression of *yheF* from an artificial promoter on the plasmid. Further investigation is required to determine the specific contributions of *hofQ* and *yheF* to the pathogenesis of CFT073.

We did not observe defects for the UTI89 or CFT073 secretin knockout mutants in adhesion, invasion, or replication in human urothelial cells. UPEC adhere to bladder and kidney cells with the help of type 1 and P pili, and in case of the bladder epithelium their entry into the superficial umbrella cells is mediated by the type 1 pilus tip adhesin, FimH [11]. Hence it was expected that none of the UTI89 or CFT073 knockout mutants, generated by specifically targeting the secretins from the putative T2SS and T4P gene clusters, would be defective in adhesion or invasion. In addition, we did not detect changes in cytotoxicity of the UPEC mutants toward host cells or ability to suppress IL-6 expression by the bladder epithelial cells. Thus, these functions in UPEC presumably are not mediated by the putative T2S and T4P pathways. Using a modified gentamicin protection assay that mimics several aspects of UPEC pathogenesis, we did observe that knockout mutants in UTI89 lacking *hofQ* and *gspD* were defective in fluxing out of the bladder epithelial cell line. This efflux defect could underlie the in vivo defect observed for the UTI89 Δ*hofQ* mutant, but we do no believe this to be the case, as the UTI89 Δ*gspD* mutant had no in vivo phenotype despite being equally defective for efflux in vitro. Furthermore, no efflux defect was found for any of the CFT073 secretin mutants. Efflux defects for the UTI89 Δ*hofQ* and Δ*gspD* mutants were confirmed by fluorescence microscopy using green fluorescent protein-expressing strains, but these experiments indicated a delay in efflux rather than an absolute defect (R.K. and D.G.T. unpublished results). This, together with the relatively small (∼2-fold) scale of the defect, could explain the lack of correlation between the cell culture and in vivo results.

Based on our findings, and assuming that the putative T2SS and T4P gene clusters function as expected based on their homology to established systems, we propose that T4P aid in the colonization and persistence of UPEC in the urinary tract, particularly the upper urinary tract. Although, flagella have been implicated in the ascent of UPEC along the ureters [42], the role for other bacterial factors that might aid in this process is still undefined. In a number of Gram-negative pathogens, association with the host epithelium followed by motility and dispersal over the epithelial surface are mediated by T4P [43]. T4P in UPEC might play a similar role, although colonization of the urinary tract at early time points post-infection were similar in both the mutant and WT strains. Alternatively, T4P may specifically aid in the colonization of kidney tissues by UPEC, possibly by mediating bacterial-bacterial and bacterial-host interactions and/or the formation of biofilms. Another possibility is that in the absence of functional T4P and/or the T2SS, the mutant bacteria were defective due to the lack of an unknown secreted effector, leading to clearance of the bacteria by day 7 post-infection. ExPEC employ a broad array of virulence factors, including type 1 and P pili, and secreted toxins such as CNF1, hemolysin and Sat. Our results suggest this impressive list of weaponry may need to be expanded to include T4P and possibly also the T2SS, opening new avenues to understanding the molecular mechanisms of *E. coli* pathogenesis in the upper urinary tract.

## Materials and Methods

### Bacterial Strains and Cell Lines

UPEC strain CFT073 was isolated from the blood of a woman suffering from pyelonephritis [35] and UPEC strain UTI89 was isolated from a cystitis patient [36]. The bacteria were grown on LB agar plates or in LB medium. When needed, the medium was supplemented with appropriate concentrations of antibiotics: 100 µg/ml ampicillin (Amp), 50 µg/ml kanamycin (Kan) and/or 25 µg/ml chloramphenicol (Clm). Human bladder (5637, ATCC # HTB-9) and human kidney (A498, ATCC # HTB-14) cell lines were obtained from the ATCC. 5637 cells were maintained in RPMI medium supplemented with 10% fetal bovine serum (FBS; Gemini). A498 cells were maintained in modified Eagle's medium (MEM) supplemented with 10% FBS, 1× sodium pyruvate and 1× l-glutamine. For growth and during infection assays, the cell lines were kept at 37°C in the presence of 5% CO_2_.

### Enumeration of Bacteria

Infected cell lines or organs were treated with 0.4 or 0.02% triton X-100 in sterile PBS, respectively. The cell monolayers were then kept at 4°C for 10 min to help lift the cells off the plastic, while the organs were homogenized using Tenbroeck tissue grinders (Kontes). The suspensions were then vortexed, serially diluted in sterile LB broth and 100 µl of appropriate dilutions were spread on LB agar plates. After incubating the plates at 37°C overnight, single colonies were counted and CFU/ml or CFU/g of organ weight calculated.

### Generation of knockout mutants

The method described by Datsenko and Wanner [39] was used to generate genomic deletion mutations in the genes coding for OM secretins, using the knockout primers listed in Table 1. In addition, primers flanking the target genes were designed (Table 1) to confirm proper occurrence of each step in the procedure by PCR. To generate double knockout mutants, one antibiotic resistance gene (for example, Kan) was first used to replace one of the target genes by homologous recombination. A different antibiotic resistance gene (for example, Cml) was then used to replace the second target gene. The resultant recombinant bacteria were screened for disruption of the two different target loci by growing on LB plates supplemented with kanamycin and chloramphenicol. Finally after introduction of plasmid-borne FLP recombinase, colonies were screened for loss of resistance to both antibiotics. Proper occurrence of each step in the procedure by was verified by PCR.

### Generation of Rescue Plasmids

The genes *hofQ* and *yheF* were PCR amplified using cloning primers listed in Table 1. The PCR products were gel purified, ligated into pGEM T-easy vector (Promega) as per the manufacturer's instructions and the ligation products were transformed into DH5α. Colonies containing pGEM-*hofQ* and pGEM-*yheF* were purified, the plasmids isolated and analyzed by DNA sequencing. The EcoRI digested fragment from pGEM-*hofQ* and the BamHI, HindIII digested fragment from pGEM-*yheF* were then ligated into pMMB67HE [44] pretreated with the same restriction endonucleases to obtain plasmids, p*hofQ* and p*yheF*. The correct orientation of the *hofQ* gene in pMMB67HE was confirmed by the DNA sequencing. These plasmids were then transformed into appropriate deletion mutant strains for use in the rescue experiments. Analysis of bacteria recovered from the mouse infection experiments demonstrated that the rescue plasmids were stably maintained in the absence of antibiotic selection (data not shown).

### Mouse Infections

Bacteria were grown statically in 20 ml LB broth at 37°C for 24–48 h. On the day of animal infection, the bacteria were centrifuged and pellets resuspended in sterile PBS to ∼10^9^ CFU/ml. 6–8 week old CBA/J female mice were anesthetized using isoflurane and 50 µl of the bacterial suspension or sterile PBS (as a negative control) was delivered to the bladder by transurethral catheterization using UV sterilized 0.28 mm inner diameter tubing (Becton Dickinson) mounted on 30½ G needle tips. Dilutions of all inocula were plated on LB agar to determine input CFU/ml. Five-to-seven mice were infected with each bacterial strain. On 1 or 7 days post-inoculation, the animals were killed by CO_2_ asphyxiation, dissected and organs (bladders and kidneys) harvested. Bladders and right side kidneys from 5 infected mice were homogenized in sterile PBS containing 0.02% triton-X 100, and serial dilutions were plated on LB agar plates. Each mouse experiment with the UTI89 and CFT073 knockout mutants was repeated at least once. Comparison of the UTI89 WT, Δ*hofQ*, and Δ*hofQ*/p*hofQ* strains was performed once. Comparison of the CFT073 WT, Δ*yheF* Δ*hofQ*, Δ*yheF* Δ*hofQ*/p*yheF* and Δ*yheF* Δ*hofQ*/p*hofQ* strains was performed twice. All animal research protocols were approved by the Institutional Animal Care and Use Committee of Stony Brook University.

### Cell Culture Infection Assays

5637 human bladder (ATCC # HTB-9) or A498 Human kidney (ATCC # HTB-14) cell lines were infected with appropriate bacterial strains in a modified gentamicin protection assay designed to assess differences between knockout mutants and their WT counterparts in one or more cardinal steps of UTI, namely adhesion, invasion, intracellular replication and efflux. The assay is described in detail in [11]. In short, confluent monolayers of appropriate cell lines were grown in 24 well tissue culture plates. Bacteria, grown statically at 37°C for 48 h were normalized so as to achieve a multiplicity of infection (MOI) of 6–8. The initial count (IC, equals total input CFUs) of bacteria was determined. To facilitate bacterial contact with the host cells, centrifugation (800×g) was carried out for 5 min. After incubation for 2 h, the cells in one set of wells were washed with PBS containing 0.9 mM Mg^2+^ and 0.49 mM Ca^2+^ (Gibco), and bacterial CFUs enumerated to give the adherent bacteria (AB; extracellular adherent bacteria plus any intracellular bacteria). Bacteria in another set of wells were enumerated without the washing step to give the total bacteria (TB). The percent adherence frequency was determined as (AB÷TB)×100. A third set of wells was treated with 100 µg/ml gentamicin for 2 h to kill all extracellular bacteria. These were then washed with PBS (Ca^2+^/Mg^2+^) five times, and the intracellular bacteria (IB) enumerated. The percent invasion frequency was determined as (IB÷IC)×100.

To measure efflux, the infection steps were carried out as described above except that after the 2 h treatment with 100 µg/ml gentamicin, the infected monolayers were washed with PBS (Ca^2+^/Mg^2+^) once and were incubated in presence of 10 µg/ml gentamicin overnight. On day 2, the intracellular bacteria in one set of wells were enumerated after washing off the gentamicin to give overnight intracellular bacteria (OB). The other two sets of wells were washed 5 times with PBS (Ca^2+^/Mg^2+^) and incubated for 6 h either in presence or absence of gentamicin. In the absence of gentamicin, the intracellular bacteria form IBCs and start effluxing out of the infected cells. The total number of bacteria in each well was enumerated to give effluxed bacteria (EB; effluxed plus intracellular bacteria). The efflux index was calculated as (EB÷OB)×100.

### IL-6 Suppression Assay

IL-6 suppression assays were carried out as described by Hunstad *et al* [40]. Briefly, 24 h old static cultures of bacteria grown at 37°C were used to infect confluent monolayers of 5637 bladder cells grown in 24 well plates. The bacterial suspension used for infection contained 5 µg/ml of O157 LPS (Sigma). Bacterial contact with cells was expedited by centrifuging the plates at 400×g for 3 min and the plates were then incubated at 37°C in presence of 5% CO_2_ for 12 h. At the end of the incubation period, the supernatant fractions were collected, spun at 13,000×g for 5 min to pellet bacteria and cell debris, and stored at −80°C until IL-6 estimation by using the human IL-6 ELISA kit (Antigenix America).

### Cytotoxicity Assay

To measure cytotoxicity during bacterial efflux, 5637 human bladder cells were infected with WT or knockout UPEC strains for an efflux assay as described above. Upon removal of gentamicin on the second day of the infection, the supernatant fractions were collected at 2, 4 and 6 h time points. These fractions were tested for the presence of lactate dehydrogenase (LDH) using the Cytotox 96 non-radioactive cytotoxicity assay (Promega). To test for instant cytotoxicity, LDH release was determined after infecting 5637 cells for 3 h with WT or knockout UPEC strains at an MOI of 5 or 10. The percent cytotoxicity was calculated as per manufacturer's instructions.

### Analysis of the T2SS and T4P gene clusters

Percent identities of protein sequences encoded by different T2SS and T4P genes were assessed using the ClustalW alignment tool from the Macvector 9.0.1. software program. Values are reported as percent of entire protein sequence.

### Statistical Analysis

The CFU/g of organ weight results from the mouse infection experiments were compared using the Mann-Whitney test for non-parametric data with one-tailed P value. The cell culture assay indices (efflux, adhesion, and invasion) were compared using Dunnett's multiple comparisons test. P values <0.05 were considered significant.
